# Farm size and biosecurity measures associated with *Strongylus vulgaris* infection in horses

**DOI:** 10.1111/evj.14212

**Published:** 2024-08-22

**Authors:** Ylva Hedberg Alm, Eva Tydén, Frida Martin, Jessica Lernå, Peter Halvarsson

**Affiliations:** ^1^ Department of Animal Biosciences, Parasitology Unit Swedish University of Agricultural Sciences Uppsala Sweden

**Keywords:** blood worm, deworming, farm management, horse, horse parasites, large strongyles

## Abstract

**Background:**

Selective anthelmintic treatment, advocated due to evolving anthelmintic resistance, has been associated with an increase in *Strongylus vulgaris* prevalence. Reverting to routine interval anthelmintic treatments is not viable and therefore, identifying other management factors correlated with *S. vulgaris* infection is vital.

**Objectives:**

To investigate possible risk factors associated with the presence of *S. vulgaris* infection in resident horses on Swedish horse establishments.

**Study design:**

Internet‐based questionnaire survey.

**Methods:**

A questionnaire, created using the internet‐based survey platform Netigate, was distributed to owners of equine establishments throughout Sweden via established equine platforms and social media channels. The survey was available for response from 21 May until 1 September 2022. Questions were closed ended with branching logic paths.

**Results:**

Four factors were significantly associated with *S. vulgaris* infection, with an increased odds of infection seen in livery yards (odds ratio [OR] 1.67, 95% confidence interval [CI] 1.18–2.36, *p* = 0.004) and premises with more than 10 resident horses (OR 2.42, 95% CI 1.64–3.56, *p* < 0.001). A lower odds of infection were seen in establishments using quarantine routines (OR 0.69, 95% CI 0.50–0.96, *p* = 0.03) and anthelmintic treatment of new horses prior to arrival at the premise (OR 0.37, 95% CI 0.18–0.74, *p* = 0.005).

**Main limitation:**

Due to the presence of *S. vulgaris* infection in the present study being based on *S. vulgaris* diagnostics performed at the farm level, any association between faecal diagnostic use and risk of infection could not be investigated.

**Conclusions:**

Although the use of diagnostics for *S. vulgaris* can keep infection rates low, large farms or livery yards with many different horse owners, and those with low use of biosecurity measures as regards to new horses arriving at the premise, are associated with a higher risk of infection.

## 1 INTRODUCTION

As grazing animals, horses are inadvertently exposed to intestinal parasites, with the majority of horses infected to some degree with cyathostomins.^1^, ^2^, ^3^, ^4^ However, although considered ubiquitous in horses with pasture access, clinical disease associated with cyathostomins is fortunately rare in well‐managed horse populations.^5^, ^6^ In contrast, *Strongylus vulgaris*, one of the large strongyles, has considerably greater pathogenicity, and thrombo‐embolic disease with non‐strangulating intestinal infarction caused by this parasite often has a fatal outcome.^7^, ^8^, ^9^ As a result of regular interval treatments with anthelmintic drugs, the prevalence of *S. vulgaris* in Sweden, as in most parts of the world, was radically reduced from 40% and 60% in 1979 to a mere 5% in the 1990s.^10^, ^11^ However, due to the emergence of anthelmintic drug resistance, selective treatment, that is, only treating certain horses based on individual faecal egg counts, often those excreting greater than 200 eggs per gram faeces, is recommended.^12^, ^13^, ^14^ Such regimes can greatly reduce the amount of anthelmintic drugs used, without significantly increasing parasite pasture contamination caused by cyathostomins.^15^ As regards to *S. vulgaris*, however, specific diagnostics are required for detection, and both Denmark and Sweden, two countries that have strong adherence to anthelmintic treatment based on faecal diagnostics, have seen a recent increase in its prevalence.^16^, ^17^ To this end, infection with *S. vulgaris* has been shown to be associated with the use of a selective anthelmintic treatment strategy, as opposed to regular treatment of all horses.^16^, ^18^ Furthermore, Tydén et al. demonstrated that excluding specific diagnostics for *S. vulgaris* was associated with an increased risk of infection.^17^ However, other specific risk factors associated with *S. vulgaris* infection have yet to be determined. Since regression to regular interval treatment with anthelmintic drugs is not acceptable, alternative methods of reducing the risk of large strongyle infection in horses are crucial. Identifying specific risk factors for infection will assist in developing strategies other than regular anthelmintic treatment to protect horses from *S. vulgaris* infection. Using an internet‐based questionnaire survey, the aim of the present study was therefore to investigate possible risk factors associated with the presence of *S. vulgaris* infection in resident horses on Swedish horse farms.

## 2 MATERIALS AND METHODS

A questionnaire designed on the internet‐based survey platform Netigate (netigate.net) was distributed as an internet link made available for response from 21 May until 1 September 2022 on specific nationwide equine orientated websites (tidningenridsport.se, hastsverige.se, hippson.se) (Table S1). In addition, awareness of and access to the questionnaire was achieved through social media channels, distributed by the authors directly, and by the proprietors of the equine websites named above, after contact with the authors. The target population was owners or managers of Swedish equine premises with adequate knowledge to be able to respond to the questions regarding all resident horses at their establishment, as opposed to individual horse owners. All questions apart from one, regarding the equine premises' postal code, were closed ended with pre‐determined answer choices. Some questions were connected by branching logic, where certain answers opened up new questions, in order for the respondent to only face relevant queries. Prior to distribution, a test version of the questionnaire was sent to 10 people with professional equine backgrounds, for control of time for completion and evaluation of the questions' clarity.

## 3 DATA ANALYSES

The questionnaire data were analysed using a generalised linear additive model (GLM) in R v4.3.1 using *S. vulgaris* findings as response variable.^19^ The full GLM contained 18 factors that were removed stepwise until only significant factors remained. To investigate independence of the questions regarding anthelmintic treatment of new horses and quarantine routines of new horses, these two factors were also evaluated for interaction effects in a GLM. Odds ratio (OR) was then calculated for significant variables using package autoReg v0.0.3 and visualised with ggplot2 v 3.4.4. with the level of significance set at 0.05.^20^, ^21^ The margin of error (precision) of the study, given a 95% confidence interval (CI), was calculated using the formula:

$$\text{Margin of error}=z\times \sqrt{\frac{p\times \left(1-p\right)}{n},}$$

where *z* is the *z*‐score associated with the CI (here 1.96), *p* is the sample proportion (we used 0.5 as it is the most conservative model) and *n* the number of respondents. Odds ratio for number of *S. vulgaris* diagnosed horses on the farm versus farm size category was calculated on the contingency table using the function ‘oddsratio’ from the package epitools v0.5.10.^22^

## 4 RESULTS

### 4.1 Questionnaire data

#### 4.1.1 *S. vulgaris* diagnostics

**FIGURE 1:**
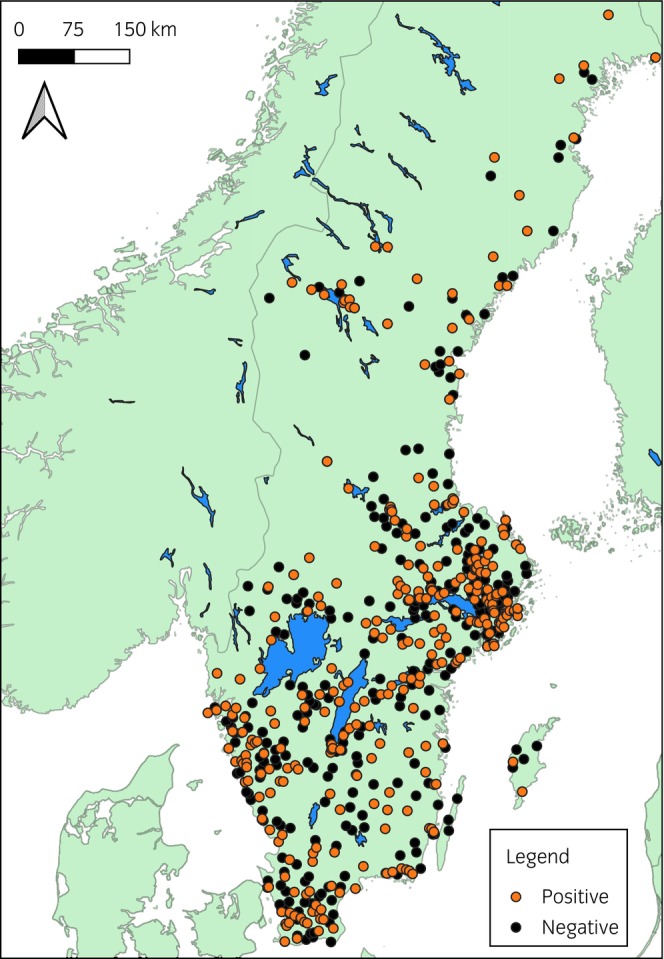
Map depicting the included establishments' geographical location in Sweden. Made with Natural Earth vector data.

The questionnaire was completed in full by a total of 1118 respondents, all of which were owners or persons responsible for the care of an entire equine premise and able to answer questions regarding their premise as a whole. This corresponds to approximately 2% of all horse farms in Sweden (67 100 ± 4100).^23^ The margin of error (precision) was calculated to be 2.93%, given a 95% CI and simple random sampling from this potential respondent population. Of the 1118 respondents, 378 (34%) did not use regular diagnostic tests for *S. vulgaris* (i.e., strongyle larval culture or real‐time PCR on individual faecal samples), precluding knowledge of possible presence of infection on their establishment, and were therefore not included for further analysis. Another 23 respondents were excluded because, despite the use of regular diagnostics for *S. vulgaris*, they declared a lack of knowledge of the presence of infection on their premise. Out of the remaining 717 respondents, 335 reported to have had at least one horse infected with *S. vulgaris* over the past 24 months, with the remaining 382 respondents declaring no horse positive for *S. vulgaris* during that same time period, that is, 47% of the farms using specific diagnostics for the parasite had detected the infection. Of the farms that reported to have positive horses for *S. vulgaris*, the number of positive horses detected over the previous 2 years varied as follows: 42% one horse, 29% two horses, 18% three to four horses and 6% more than four horses. In 5% of farms, the number of positive horses was unknown. There was a successive increase in the number of farms with more than 10 resident horses with the number of horses detected positive for *S. vulgaris*, with one, two, three to four and more than four positive horses detected, associated with 28%, 38%, 49% and 64% of the farms having more than 10 resident horses, respectively. The geographical distribution of all included farms is depicted in Figure 1. The majority of premises were located in the southern half of Sweden, which corresponds to the most horse‐dense areas of Sweden.^22^

#### 4.1.2 Anthelmintic routines

**FIGURE 2:**
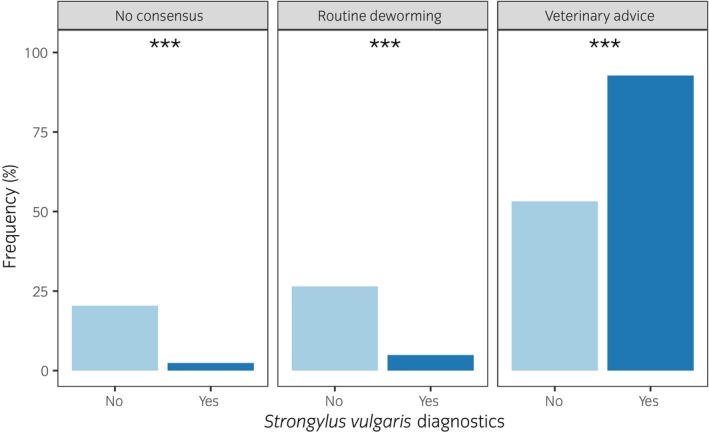
Anthelmintic routines (i.e., routine treatment, treatment based on veterinary advice and/or positive faecal samples or no unified consensus/unknown treatment routine) used by premises using regular diagnostics for *Strongylus vulgaris* (*n* = 717) and premises not using *S. vulgaris* diagnostics (*n* = 378). Significant differences were found between the two groups for all specified anthelmintic routines (*p* < 0.001).

Although excluded from the risk assessment analysis, due to the presence of *S. vulgaris* infection over the past 24 months being unknown, there were significant differences noted regarding which anthelmintic routines were employed on premises that used *S. vulgaris* diagnostics (i.e., individual faecal samples for either larval culture or real‐time PCR) (*n* = 717) and those that did not (*n* = 378), as depicted in Figure 2 (*p* < 0.001). Notably, there was less veterinary involvement and a greater use of routine treatments on establishments that did not use regular extended diagnostic tests for *S. vulgaris*. Furthermore, it was more common for these premises to have no established unified treatment regime.

#### 4.1.3 General description of included premises and management routines

A general description of all establishments using extended diagnostics for *S. vulgaris*, including premise type, farm‐size and horse‐turnover is shown in Table 1. For a description of anthelmintic routines and management of new arrivals on these premises, see Table 2. Response to questions regarding type of housing and pasture access is depicted in Table S2, with response to questions regarding pasture management shown in Table S3.

**TABLE 1:** Response to questions regarding establishment type and size, including number of new arrivals, expressed as a percentage of the total number of responses given by respondents from premises that had diagnosed *Strongylus vulgaris* positive horses (*n* = 335) and by respondents from premises without positive horses (*n* = 382).

Question and response alternatives	Response by *S. vulgaris* positive farms (%)	Response by *S. vulgaris* negative farms (%)
Type of premise		
Livery stable	72.5	54.2
Racing stable	2.4	1.0
Competition stable	11.0	9.4
Stud farm	10.4	11.0
Riding school	6.0	3.7
Horse sale	4.5	1.8
Horse trekking	0.6	1.0
Horse rental	0.9	0.8
Other	26.9	29.4
Number of resident horses		
1–5	39.7	57.9
6–10	22.0	23.3
11–15	14.9	6.3
16–25	13.4	9.4
>25	9.9	3.1
Number of new arrivals past 12 month		
0	23.2	31.4
1–5	62.7	62.3
6–10	11.6	5.2
>10	2.4	1.0

**TABLE 2:** Response to questions regarding anthelmintic routines and management of new arrivals, expressed as a percentage of the total number of responses given by respondents from premises that had diagnosed *Strongylus vulgaris* positive horses (*n* = 335) and by respondents from premises without positive horses (*n* = 382).

Question and response alternatives	Response by *S. vulgaris* positive farms (%)	Response by *S. vulgaris* negative farms (%)
Anthelmintic treatment routine		
Routine treatment 1–4/year	3.3	6.3
Selective treatment based on faecal samples or as directed by veterinarian	95.8	90.1
No consensus or unknown	0.9	3.7
Treatment when *S. vulgaris* positive horses are detected (only farms with positive horses)^a^		
All horses sharing same pasture	39.1	N/A
All horses at the establishment	38.2	N/A
Only positive horse(s)	22.7	N/A
Treated once	25.4	N/A
Treated more than once	74.6	N/A
Treatment of new arrivals		
If indicated by faecal sample	43.3	40.6
Always	24.8	30.9
Never	10.1	6.3
Sometimes	11.9	4.7
Prior to arrival	9.9	17.0
Quarantine of new arrivals		
Yes, for 1–2 weeks	40.9	49.2
No	59.1	50.8

^a^ Only premises with positive *S. vulgaris* horses during the past 24 months, more than one alternative possible.

### 4.2 Associations between questionnaire data and the presence of *S. vulgaris* infection

**FIGURE 3:**
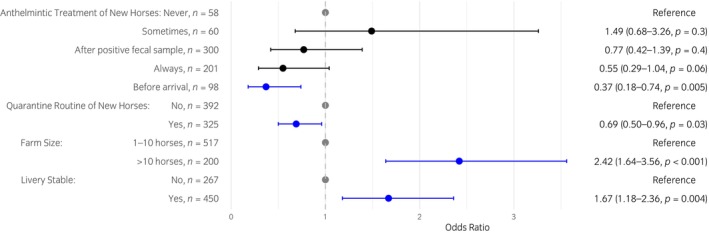
Odds ratio of the four variables significantly associated with *Strongylus vulgaris* infection based on 717 responses. For question regarding anthelmintic treatment of new horses, the response ‘never’, answered by 58 responders, was set as the reference value. Significant response alternatives for each variable are depicted in blue and the response used as a reference in grey. Level of significance was set at 0.05.

In total, four factors were significantly associated with the presence of *S. vulgaris* infection on the farm. Of these, two were farm‐related, whereas the other two factors were related to management practices of new horses arriving at the premise. As such, farm size was significantly associated with the risk of having had at least one horse positive for *S. vulgaris* within the previous 24 months (*p* < 0.001), with 2.42 times (95% CI 1.64–3.56) higher odds of infection on large premises (>10 horses), compared with premises with 10 or fewer horses (Figure 3). The odds ratio of a farm having more than 10 resident horses showed a significant and successive increase with the number of positive horses detected on the farm, as depicted in Table 3. Furthermore, the presence of *S. vulgaris* infection was significantly associated with premise type, with 1.67 times (95% CI 1.18–2.36) higher odds of infection in livery stables compared with other types of equestrian establishments (*p* = 0.004). Using quarantine of new horses arriving at the premise was associated with a significantly lower odds of infection (OR 0.69, 95% CI 0.50–0.96, *p* = 0.03). In addition, anthelmintic treatment of new horses prior to arrival was associated with a decreased odds (OR 0.37, 95% CI 0.18–0.74) of *S. vulgaris* positive horses being present on the farm (*p* = 0.005). No interaction between using quarantine of new horses arriving at the premise and anthelmintic treatment of new arrivals could be detected based on a GLM of these two factors (*p* > 0.05).

**TABLE 3:** Odds ratio (OR) estimates and 95% confidence intervals (CIs) for number of horses diagnosed positive for *Strongylus vulgaris* on farms with 1–10 horses compared with farms with more than 10 horses.

Number of positive horses vs. farm size	OR estimate	Lower CI	Upper CI	*p*‐value	*N*
0 horses	1	–	–	Reference	382
1 horse	1.71	1.08	2.66	0.022	141
2 horses	3.14	1.94	5.07	<0.001	97
3–4 horses	4.01	2.26	7.11	<0.001	60
>4 horses	7.26	2.79	20.46	<0.001	19
Do not know	2.17	0.72	5.87	0.13	18

*Note*: Farms with horses diagnosed positive for *S. vulgaris* had a higher odds ratio to be larger farms with more than 10 horses irrespectively on how many tested positive as indicated by the *p*‐value (Fishers exact test). The 382 farms that did not have a horse diagnosed positive for *S. vulgaris* was used as the reference.

## 5 DISCUSSION

Sweden and Denmark are unique countries in that the majority of equine owners perform anthelmintic treatment of their adult horses based exclusively on faecal sample results.^16^, ^17^, ^24^ Furthermore, as shown in the present study, specific diagnostics for *S. vulgaris* using PCR technique or morphological species determination on strongyle larval cultures from individual faecal samples is commonly used.^17^, ^24^ This extensive use of faecal diagnostics and consequent knowledge of current and historic presence of parasite infection on equine premises allows for the use of survey‐based studies. As such, using an internet‐based questionnaire, we demonstrated farm‐related risk factors associated with the presence of *S. vulgaris* infection in horse farms in Sweden. Specifically, large premises and livery stables were correlated with a greater risk of infection. Additionally, management practices concerning new horses arriving at a farm influenced the presence of infection, with the use of quarantine of new horses after arrival and treating new horses with an anthelmintic drug prior to arrival at the farm associated with a lowered risk.

To date, most studies investigating risk factors associated with intestinal parasite infection in horses have focused on cyathostomins and *Parascaris* spp. Infection rates in these parasites have shown a strong association with age, with young individuals more prone to infection and high parasite burdens.^24^, ^25^, ^26^, ^27^, ^28^ In addition, pasture access is significantly correlated with cyathostomin infection.^28^, ^29^, ^30^ In this context, one study showed that both young age and increase in pasture access were associated with an increase in both strongyle egg excretion and the presence of *S. vulgaris* antibodies.^18^ In agreement, Stoughton et al. found that racehorses had significantly lower odds of having a positive titre to *S. vulgaris*, speculated to be due to more limited grazing time compared with non‐racehorses.^31^ However, purposefully restricting access to grazing is not an appropriate measure to lower infection risk, whereby studies exploring other management or farm related factors associated with the risk of *S. vulgaris* infection are needed.

Our results showed an association between the presence of *S. vulgaris* infection at the farm level and large equine establishments and livery stables. Similarly, *S. vulgaris* infection in donkeys was shown to be significantly associated with herd size, with increasing infection rates in herds with more than 50 animals.^32^ Large farm size has also been linked to an increased prevalence of *Parascaris* infection in young horses, thought to be a result of higher infection pressures and a greater risk of anthelmintic resistance.^33^ Although anthelmintic resistance in *S. vulgaris* as yet has not been reported, a greater infection pressure associated with a larger number of resident horses is a possible explanation for the increased risk of *S. vulgaris* infection observed in the present study. Furthermore, most livery stables in Sweden can be described as DIY yards, where each owner cares for their own horse, with shared pastures and other facilities. Thus, speculatively, despite veterinary involvement and regular faecal testing, the increased odds risk of *S. vulgaris* infection demonstrated on such yards may be related to a lack of consensus regarding anthelmintic routines, such as timing of faecal samples and anthelmintic treatments. In addition, it could be considered that larger premises and livery yards may have less than optimal pasture management routines and higher stocking densities, compared with smaller establishments. However, the present study found no association between pasture management routines, stocking densities and the presence of *S. vulgaris* infection.

Farms that diagnosed horses to be positive for *S. vulgaris* were more likely to be larger farms with more than 10 horses irrespectively of how many horses tested positive. However, it is acknowledged that, considering the overall low number of positive horses detected, there is a possibility that the smaller farms did not detect positive horses, simply because fewer horses were tested. Individual faecal samples were used by all owners, but despite this, it is recognised that false negative results can occur, particularly in farms using morphological larval culture differentiation, which was shown to have a sensitivity of 73% (and a specificity of 84%) compared with necropsy data.^34^ Real‐time PCR has higher sensitivity than larval cultures, with a detection limit less than or equal to 0.5 *S. vulgaris* eggs, as well as an indication of 100% specificity.^35^, ^36^

In the present study, treating horses with an anthelmintic drug before arrival was associated with a lower odds of *S. vulgaris* infected horses at the establishment. Treating horses prior to arrival could involve a risk of horses being re‐infected at the existing premises, and therefore it is somewhat surprising that this strategy, as opposed to treating at arrival, appeared to be the most favourable strategy. However, it is appreciated that the questionnaire did not allow for further specification as to how horses receiving an anthelmintic drug prior to arrival were managed after treatment; for example, the use of separate gravel paddocks or similar could reduce the risk of re‐infection. Further, usage of quarantine practices for new arrivals lowered infection risk. By not introducing new horses to a shared pasture immediately at arrival, time is given to treat the horses with an anthelmintic drug without the risk of prior pasture contamination. No significant interaction was found between anthelmintic treatment of new arrivals and the use of quarantine measures, suggesting quarantine, as opposed to simply treating new horses without separation from the resident horses, is important in reducing the risk of *S. vulgaris* infection. Furthermore, previous studies have shown that applying biosecurity measures when introducing new horses also decreases the risk of introducing resistant parasites.^37^, ^38^, ^39^

Somewhat surprisingly, no pasture management method in the present study was found to be significantly associated with the presence of *S. vulgaris* infection. Thus, the present study suggests that, at low infection levels and with regular *S. vulgaris* diagnostics, pasture management does not appear to have a major influence on the risk of *S. vulgaris* infection. However, the results nonetheless point to further potential for reducing parasite infection pressures. For example, similarly to what has been shown in previous surveys, only a minority of premises in the present study declared to use regular faecal removal in the summer.^24^, ^40^, ^41^, ^42^, ^43^ This is regrettable, given that faecal removal twice weekly has been shown to be highly effective in reducing parasite infection pressures.^43^, ^44^, ^45^ Furthermore, other management practices to reduce parasite burdens, such as resting pastures, ploughing or rotational grazing with another species, were only employed by a minority of the included farms. One possible explanation for this lack of pasture management could be that the majority of equestrian premises in Sweden are located in urban regions, with limited access to grazing, making resting or ploughing of pastures prohibitive.^23^

Although it is a major concern that one third of all responders declared not to make use of regular *S. vulgaris* diagnostics, previous studies conducted in Sweden showed an even greater lack of specific diagnostic usage, suggesting that diagnostics for *S. vulgaris*, although not universal, are becoming increasingly more commonplace.^17^, ^24^ To this end, countries where national legislation enforces prescription only restrictions on anthelmintic drugs, which includes Sweden as well as Denmark and the United Kingdom, appear to be experiencing pronounced changes in anthelmintic treatment strategies, with a clear increase in adherence to current recommendations.^24^, ^46^, ^47^, ^48^

Overall, the number of *S. vulgaris* positive horses over the past 2 year period was low, with only one to two positive horses detected on the majority of farms that had the infection. A recently published study, presenting data from the Swedish Veterinary Institute's parasite monitoring programme during the years 2008–2017, showed between 4% and 11% of horses to be positive for *S. vulgaris*.^15^ A substantially higher occurrence was found in the study performed by Tydén et al., where 28% of all tested horses were positive for *S. vulgaris*.^17^ A major difference between our study and that of Tydén et al. was that the present study was based purely on questionnaire data. Thus, the infection rate on the farms that did not use regular diagnostics for *S. vulgaris* (34%) was unknown and these farms had to be excluded from further analyses. Considering that Tydén et al. demonstrated a 2.9 higher odds of infection in farms not using diagnostic testing for *S. vulgaris*, the actual number of farms with positive horses in the present study may have been much greater.^17^ Moreover, in the present study, premises that did not use *S. vulgaris* diagnostics were less likely to base anthelmintic treatments on faecal samples and/or veterinary advice, with 27% declaring to routinely treat their horses 1–4 times per year and 20% reporting either no knowledge of which anthelmintic routines were used or a lack of consensus in a defined anthelmintic routine. In contrast, all farms using extended diagnostics, both with and without positive horses, declared high veterinary involvement and low use of routine treatment. In addition to the exclusion of farms not using specific *S. vulgaris* diagnostics, it is recognised that questionnaire surveys in general are likely to involve some degree of both selection and response biases. For instance, the present survey was distributed through nationwide equine orientated websites and social media channels, selecting for equine premise owners active on such communication platforms.^49^ Further, the topic of the survey, that is, parasite management, could select for respondents with a special interest in parasite control strategies. Thus, future studies combining questionnaire data with faecal samples and serology for detecting *S. vulgaris* infection are needed to fully elucidate risk factors for infection, including diagnostics and treatment routines.

In conclusion, our study supports the use of diagnostics for *S. vulgaris* to keep infection rates low. However, infection can still occur, despite regular faecal diagnostic tests for the parasite, primarily on large farms or livery yards with many different horse owners, and those with low use of biosecurity measures as regards to new horses arriving at the premise.

## FUNDING INFORMATION

The Foundation for Swedish and Norwegian Equine Research, grant number H‐15‐47‐097.

## CONFLICT OF INTEREST STATEMENT

The authors have declared no conflicting interests.

## AUTHOR CONTRIBUTIONS

**Ylva Hedberg Alm:** Conceptualization; investigation; writing – original draft; visualization; writing – review and editing; validation; project administration; supervision; formal analysis; data curation; methodology. **Eva Tydén:** Conceptualization; funding acquisition; investigation; methodology; validation; writing – review and editing; project administration; resources; supervision. **Frida Martin:** Writing – review and editing; validation. **Jessica Lernå:** Investigation; methodology; writing – review and editing; formal analysis. **Peter Halvarsson:** Software; formal analysis; data curation; validation; methodology; visualization; writing – review and editing; resources.

## DATA INTEGRITY STATEMENT

Ylva Hedberg Alm, Eva Tydén and Peter Halvarsson are responsible for data integrity and the accuracy of the data analysis.

## ETHICAL ANIMAL RESEARCH

Ethical review and approval were waived for this study in accordance with relevant guidelines and regulations issued by the Swedish Board of Agriculture's regulations and general advice on laboratory animals (SJVFS 2019:9, case no. L150).

## INFORMED CONSENT

Explicit informed consent was given by all participants of the survey.

### PEER REVIEW

The peer review history for this article is available at https://www.webofscience.com/api/gateway/wos/peer‐review/10.1111/evj.14212.

## Supporting information


**Table S1.** Netigate questionnaire including English translation of original questions.

**Table S2.** Response to questions regarding type of housing and pasture access, expressed as a percentage of the total number of responses given by respondents from premises that had diagnosed *Strongylus vulgaris* positive horses (*n* = 335) and by respondents from premises without positive horses (*n* = 382).

**Table S3.** Response to questions regarding pasture management, expressed as a percentage of the total number of responses given by respondents from premises that had diagnosed *Strongylus vulgaris* positive horses (*n* = 335) and by respondents from premises without positive horses (*n* = 382). No significant differences in any of the responses were found (*p* > 0.05).

## Data Availability

The data that support the findings of this study are openly available in SND.gu.se (Doris) at https://doi.org/10.5878/jcmx-cm10.
