# Supplementary material for: Farm size and biosecurity measures associated with Strongylus vulgaris infection in horses
Source: Equine Vet J. 2024 Aug 22;57(3):703–11. doi: 10.1111/evj.14212 (PMC11982428; doi:10.1111/evj.14212)
Supplement: Supplementary file 1 — Table S1. Netigate questionnaire including English translation of original questions. [file EVJ-57-703-s003.pdf]

Equine Veterinary Journal BEVA

Table S1: Netigate questionnaire including English translation of original questions.

**Swedish**	**English translation**
1. Kan du besvara enkäten utifrån hur rutinerna ser ut för stallet/gården i sin helhet?<br><br>*Ja/Nej*	1. Can you answer the survey based on the routines of the premise as a whole?<br><br>*Yes/No*
2. Postnummer	2. Postal code
3. Hur många hästar finns det på gården?<br><br>*1-5/6-10/11-15/16-20/21-25/26-30/>30*	3. How many horses are there at the premise?<br><br>*1-5/6-10/11-15/16-20/21-25/26-30/>30*
4. Vilken typ av stall/verksamhet? (Flera alternativ kan väljas)<br><br>*Inackorderingsstall med flera hästägare/trav-galopp stall/tävlingsstall/stuteri-uppfödning/ridskola/tillridning-försäljningsstall/turridning/uthyrning/annat*	4. What type of stable/establishment? (Several responses can be chosen)<br><br>*Livery stable with several horse owners/racing stable/competition yard/stud-breeding farm/sales stable/horse trekking/horse rental/other*
5. Hur många nya hästar har flyttat till gården de senaste 12 månaderna?<br><br>*1-5/6-10/11-15/>15*	5. How many new horses have moved to the premise within the past 12 months?<br><br>*1-5/6-10/11-15/>15*
6. Vilket alternativ stämmer med era avmaskningsrutiner?<br><br>*Vi skickar träckprov för både äggräkning och odling stor blodmask minst en gång per år på samtliga hästar/vi skickar träckprov endast för äggräkning*<br>*minst en gång per år/vi skickar inte träckprov/alla i stallet gör olika/vet inte-annat*	6. Which alternative corresponds with your anthelmintic routine?<br><br>*We send faecal samples for both egg counts and culture of large strongyles at least once per year from all horses/we send faecal samples only for faecal egg counts at least once per year/we do not send faecal samples/everyone at the premise has different routines/do not know-other*
7. Hur avmaskas hästarna?<br><br>*Vi avmaskar endast när rådgivande veterinär anses att det behövs/vi avmaskar alltid en gång per år, oavsett träckprovsresultat/vi avmaskar alltid minst 2-4 gånger per år, oavsett träckprovsresultat/vi avmaskar positiva hästar flera gånger per år, övriga avmaskas inte/vet ej-annat*	7. How are the horses dewormed?<br><br>*We only deworm when consulting veterinarian deems necessary/we always deworm once per year, regardless of faecal test result/we always deworm 2-4 times per year, regardless faecal test result/we deworm all positive horses several times per year, others are not treated/do not know-other*
8. Har någon häst i stallet haft stor blodmask (*Strongylus vulgaris*) de senaste 24 månaderna?<br><br>*Ja/nej/vet ej*	8. Has any horse tested positive for large strongyles (*Strongulys vulgaris*) within the past 24 months?<br><br>*Yes/no/do not know*
9. Hur många av hästarna var positiva?<br><br>*1/2/3-4/>4/vet ej*	9. How many horses were positive?<br><br>*1/2/3-4/>4/do not know*
10. Hur hanteras hästarna vid positivt träckprov för stor blodmask?<br><br>*Enbart de positiva hästarna avmaskas/samtliga hästar i stallet avmaskas/hästarna i samma hage som den positiva hästen avmaskas*	10. How are horses positive for large strongyles treated?<br><br>*Only the positive horses are dewormed/all horses at the premise are dewormed/horses sharing the same paddock with the positive horse is dewormed*
11. Avmaskas hästarna en gång eller upprepande gånger?	11. Are the horses dewormed once or repeated times?

En gång/upprepade gånger	*Once/repeated times*
12. Vad använder ni för typ av inhysningssystem?<br><br>*Spiltor-boxar med daglig utevistelse/aktiv grupphästhållning (Active stable)/lösdrift*	12. What type of housing is used?<br><br>*Stable-kept with daily turn-out/active stable/grass-kept*
13. Har hästarna fasta hagar? (Bortsett från om de flyttas mellan sommar- och vinterhagar)<br><br>*Ja/nej/vet ej*	13. Do the horses keep the same turn-out paddocks? (Apart from movement between summer and winter paddocks)<br><br>*Yes/no/do not know*
14. Har ni separata vinter/sommarhagar?<br><br>*Ja/nej*	14. Do you have separate winter/summer paddocks?<br><br>*Yes/no*
15. Hur hanteras beteshagarna?<br><br>*Vila 2 vintrar + mellanliggande sommar/vila 2 somrar + mellanliggande vinter/plöjer hagarna/växelarbete med andra djurslag/har separat sommar- och vinterhage men ingen åtgärd*	15. How are the paddocks managed?<br><br>*Rest 2 winters + in between summer/rest 2 summers + in between winter/harrowing/rotational grazing with other species/separate summer and winter paddocks, but no other management*
16. Hur stor yta/häst i sommarhagen? (1 hektar = 10.000 m^2)<br><br>*Mindre än 0.5 hektar per häst/0.5-1 hektar per häst/mer än 1 hektar per häst/vet ej*	16. How large area/horse in the summer paddock? (1 hectare = 10,000 m^2)<br><br>*Less than 0,5 hectare per horse/0,5-1 hectare per horse/more than 1 hectare per horse/do not know*
17. Hur stor yta/häst i vinterhagen? (1 hektar = 10.000 m2)<br><br>*Mindre än 0.5 hektar per häst/0.5-1 hektar per häst/mer än 1 hektar per häst/vet ej*	17. How large area/horse in the winter paddock? (1 hectare = 10,000 m^2)<br><br>*Less than 0,5 hectare per horse/0,5-1 hectare per horse/more than 1 hectare per horse/do not know*
18. Hur stor yta/häst i hagen? (1 hektar = 10.000 m2) (ej separata vinter-och sommarhagar)<br><br>*Mindre än 0.5 hektar per häst/0.5-1 hektar per häst/mer än 1 hektar per häst/vet ej*	18. How large area/horse in the paddock? (1 hectare = 10,000 m^2) (not separate winter and summer paddocks)<br><br>*Less than 0,5 hectare per horse/0,5-1 hectare per horse/more than 1 hectare per horse/do not know*
19. Hur många hästar/hage under sommaren?<br><br>*1 häst per hage/2 hästar per hage/3-5 hästar per hage/fler än 5 hästar per hage/vet ej-annat*	19. How many horses/paddock in the summer?<br><br>*1 horse per paddock/2 horses per paddock/3-5 horses per paddock/more than 5 horses per paddock/do not know-other*
20. Hur många hästar/hage under vintern?<br><br>*1 häst per hage/2 hästar per hage/3-5 hästar per hage/fler än 5 hästar per hage/vet ej-annat*	20. How many horses/paddock in the winter?<br><br>*1 horse per paddock/2 horses per paddock/3-5 horses per paddock/more than 5 horses per paddock/do not know-other*
21. Hur många hästar/hage? (ej separata vinter- och sommarhagar)	21. How many horses/paddock? (not separate winter and summer paddocks)

1 häst per hage/2 hästar per hage/3-5 hästar per hage/fler än 5 hästar per hage/vet ej-annat	*1 horse per paddock/2 horses per paddock/3-5 horses per paddock/more than 5 horses per paddock/do not know-other*
22. Typ av hage på sommaren?<br><br>*Beteshage/skogsbete/ängsmark/sandpaddock/ grus/annat*	22. Type of paddock in the summer?<br><br>*Grazing/forest-woodland/meadow/sand paddock/gravel/other*
23. Typ av hage på vintern?<br><br>*Beteshage/skogsbete/ängsmark/sandpaddock/ grus/annat*	23. Type of paddock in the winter?<br><br>*Grazing/forest-woodland/meadow/sand paddock/gravel/other*
24. Vad har ni för typ av hage? (ej separata vinter- och sommarhagar)<br><br>*Beteshage/skogsbete/ängsmark/sandpaddock/ grus/annat*	24. Which type of paddock do you have? (not separate winter and summer paddocks)<br><br>*Grazing/forest-woodland/meadow/sand paddock/gravel/other*
25. Hur ofta mockas hagarna på vintern?<br><br>*Dagligen/cirka 1-2 gånger i veckan/cirka 1-2 gånger i månaden/mockas ej regelbundet*	25. How often are faeces removed from winter paddocks in the winter?<br><br>*Daily/approx. 1-2 times per week/approx. 1-2 times per month/no regular faecal removal*
26. Hur ofta mockas hagarna på sommaren?<br><br>*Dagligen/cirka 1-2 gånger i veckan/cirka 1-2 gånger i månaden/mockas ej regelbundet*	26. How often are faeces removed from the paddocks in the summer?<br><br>*Daily/approx. 1-2 times per week/approx. 1-2 times per month/no regular faecal removal*
27. Avmaskas nya hästar vid ankomst till gården/stallet?<br><br>*Ja/endast om träckprov visar på parasiter/ibland/nej/avmaskas innan ankomst till nya gården/stallet*	27. Are new horses dewormed on arrival at the premise?<br><br>*Yes/only if indicated by faecal sample/no/dewormed prior to arrival at the premise*
28. Vad har ni för karantänrutin för nya hästar?<br><br>*Hästen i box eller separat hage 1 vecka/hästen i box eller separat hage över 1 vecka/ingen karantänrutin*	28. What quarantine routine do you have for new horses?<br><br>*Horse stabled or separate paddock for 1 week/ horse stabled or separate paddock for more than 1 week/no quarantine*
