# Supplementary material for: Farm size and biosecurity measures associated with Strongylus vulgaris infection in horses
Source: Equine Vet J. 2024 Aug 22;57(3):703–11. doi: 10.1111/evj.14212 (PMC11982428; doi:10.1111/evj.14212)
Supplement: Supplementary file 2 — Table S2. Response to questions regarding type of housing and pasture access, expressed as a percentage of the total number of responses given by respondents from premises that had diagnosed Strongylus vulgaris positive horses (n = 335) and by respondents from premises without positive horses (n = 382). [file EVJ-57-703-s001.pdf]

Equine Veterinary Journal BEVA

Table S2: Response to questions regarding type of housing and pasture access, expressed as a percentage of the total number of responses given by respondents from premises that had diagnosed *S. vulgaris* positive horses (n = 335) and by respondents from premises without positive horses (n = 382). No significant differences in any of the responses were found (p > 0.05).

**Question and response alternatives**	**Response by *S. vulgaris* positive farms (%)**	**Response by *S. vulgaris* negative farms (%)**
**Type of housing**		
Loose box with daily pasture access	73.1	70.7
Active stable access	0.6	1.3
Grass-kept	26.3	28.0
**Separate summer/winter pastures/paddocks?**		
Yes	77.6	73.3
No	22.4	26.7
**Type of summer paddock/pasture**		
Grazing/meadow	86.0	87.9
Forest/woodland	8.3	10.0
Sand/gravel	1.0	0.4
Other/not stated	4.7	1.8
**Type of winter paddock/pasture**		
Grazing/meadow	51.5	55.0
Forest/woodland	18.8	20.4
Sand/gravel	17.3	13.2
Other/not stated	12.3	11.4
**Type of paddock/pasture on farms with permanent year round pastures**		
Grazing/meadow	77.3	78.4
Forest/woodland	5.3	12.7
Sand/gravel	5.3	4.9
Other/not stated	12.0	3.9
**Number of horses/paddock - summer season**		
One	5.0	4.3
Two	27.7	32.5
3-5	46.5	48.6
>5	18.1	13.6
Unknown	2.7	1.1
**Number of horses/paddock - winter season**		
One	12.3	10.0
Two	34.6	37.5
3-5	41.2	42.5
>5	9.6	9.3
Unknown	2.3	0.7
**Number of horses/paddock – permanent pasture**		
One	10.7	9.8
Two	45.3	47.1
3-5	28.0	36.3
>5	10.7	2.9
Unknown	5.3	3.9
**Stocking density - summer season**		
> 1 hectare/horse	26.9	31.8
0.5-1 hectare/horse	41.9	48.9
< 0.5 hectare/horse	20.4	15.0
Unknown	10.8	4.3
**Stocking density – winter season**		
> 1 hectare/horse	8.8	8.6
0.5-1 hectare/horse	35.4	38.2
< 0.5 hectare/horse	49.2	49.3
Unknown	6.5	4.3
**Stocking density – permanent pasture**		
> 1 hectare/horse	17.3	20.6
0.5-1 hectare/horse	50.7	53.9
< 0.5 hectare/horse	28.0	20.6
Unknown	24.0	4.9
