# Supplementary material for: Farm size and biosecurity measures associated with Strongylus vulgaris infection in horses
Source: Equine Vet J. 2024 Aug 22;57(3):703–11. doi: 10.1111/evj.14212 (PMC11982428; doi:10.1111/evj.14212)
Supplement: Supplementary file 3 — Table S3. Response to questions regarding pasture management, expressed as a percentage of the total number of responses given by respondents from premises that had diagnosed Strongylus vulgaris positive horses (n = 335) and by respondents from premises without positive horses (n = 382). No significant differences in any of the responses were found (p > 0.05). [file EVJ-57-703-s002.pdf]

Equine Veterinary Journal BEVA

Table S3: Response to questions regarding pasture management, expressed as a percentage of the total number of responses given by respondents from premises that had diagnosed *S. vulgaris* positive horses (n = 335) and by respondents from premises without positive horses (n = 382). No significant differences in any of the responses were found (p > 0.05).

Question and response alternatives	Response by *S. vulgaris* positive farms (%)	Response by *S. vulgaris* negative farms (%)
**Herd composition**		
Fixed herd composition	89.9	88.0
Movement of horses between herds	10.1	12.0
**Use of separate pastures by season**		
Yes	77.6	73.3
No	22.4	26.7
**Pasture management‡**		
Rotational grazing with other species	9.2	11.5
Ploughing of pastures	4.2	4.5
Rest of pasture two winters/one summer	4.2	1.6
Rest of pasture two summers/one winter	4.6	1.6
**Faecal removal - summer season**		
Daily	11.0	14.1
1-2 times/week	20.0	19.1
1-2 times/month	12.8	12.0
Irregularly/none	56.1	54.7
**Faecal removal - winter season**		
Daily	19.4	20.7
1-2 times/week	23.0	27.5
1-2 times/month	14.3	12.8
Irregularly/none	43.3	39.0

‡only premises with separate summer/winter paddocks
